# Morbid obesity leads to increased skin autofluorescence independent of metabolic syndrome components

**DOI:** 10.55730/1300-0144.5411

**Published:** 2022-04-02

**Authors:** Tuğçe APAYDIN, Dilek GOGAS YAVUZ

**Affiliations:** Department of Endocrinology and Metabolism, Faculty of Medicine, Marmara University, İstanbul, Turkey

**Keywords:** Advanced glycation end products, metabolic syndrome, morbid obesity, skin autofluorescence

## Abstract

**Background/aim:**

Obesity, diabetes mellitus, and metabolic syndrome (MetS) are associated with increased accumulated skin advanced glycation end products. We aimed to evaluate the association of MetS components with skin autofluorescence (SAF) in patients with morbid obesity.

**Material and methods:**

Eight hundred and one patients with morbid obesity and 94 age-matched controls with normal body mass index (BMI) and normal glucose metabolism were included. Advanced glycation end products (AGEs) were measured using SAF in the forearm, with an AGE reader.

**Results:**

The prevalence of MetS in patients with morbid obesity was 65.5% (n = 525). Type 2 diabetes mellitus (type 2 DM) and hypertension were present in 40.9% (n = 328) and 43.7% (n = 357). Patients with morbid obesity and those with MetS had higher SAF measurements compared with the control group, 1.85 ± 0.44 arbitrary unit (AU) and 1.86 ± 0.43 AU vs. 1.72 ± 0.30 AU, respectively (p = 0.016). There was no difference in SAF levels between patients with and without MetS. SAF measurements of patients without MetS were not statistically different from the control group (p = 0.076). Patients with five MetS criteria had higher SAF measurements compared with patients with fewer MetS components (p = 0.019). There was no difference in SAF levels between patients with type 2 DM, impaired glucose metabolism, and patients with normal glucose metabolism (p = 0.513).

**Conclusion:**

Although MetS and type 2 DM are known as factors related to increased SAF levels, obesity can cause elevated SAF measurements in different ways independent of concomitant comorbid diseases. Larger studies with longer follow-ups are needed to enlighten the underlying mechanism.

## 1. Introduction

Obesity is a component of metabolic syndrome (MetS) or predisposes to MetS [1], which constitutes several cardiovascular risk factors, including insulin resistance, abdominal obesity, atherogenic dyslipidemia, and hypertension, and its prevalence has been increasing due to the growing epidemic of obesity and type 2 diabetes mellitus (type 2 DM) [2]. Although patients with obesity are predisposed to MetS, it does not develop in all patients with obesity [1].

The obesity-related impaired endothelial function has been reported in previous reports, which was evaluated via measurement of carotid intima-media thickness, flow-mediated endothelial-dependent vasodilation, advanced glycation end products (AGEs), the receptor of AGEs, and skin autofluorescence (SAF) [3–6]. AGEs are glycated proteins, lipids, and nucleic acids that present with chronic exposure to hyperglycemia, inflammation, and oxidative stress [3, 7]. AGEs are considered a marker of metabolic memory [5, 8]. AGEs can accumulate in tissues through aging, dietary intake, and smoking. Studies over the last decade have revealed a progressive role of AGEs in the development of type 2 DM, cardiovascular disease (CVD), hypertension, and other chronic diseases [9, 10].

Measurement of skin AGE accumulation using autofluorescence correlates with the AGEs in skin biopsies such as pentosidine, carboxymethyl lysine (CML), and carboxyethyl lysine (CEL) [7,10]. The relationship between micro-macrovascular complications and skin autofluorescence in patients with diabetes mellitus, renal failure, and cardiac diseases has been previously investigated [8,9,11,12], and SAF is an independent predictor of long-term cardiovascular complications. However, limited studies reported AGEs levels [13–15] or SAF [5, 16] in patients with obesity. Body mass index (BMI) was found to be inversely correlated with SAF in patients with obesity [5]. Increased AGEs and decreased soluble AGE receptors were detected in patients with obesity [5,17,18].

In previous reports [3,19], patients with MetS had higher SAF measurements compared with subjects without MetS. Also as the components of MetS increased, elevated SAF values were detected [19]. The decreased soluble form of receptor for advanced glycation end products (sRAGE) [20] and increased serum AGEs [13] were also found to be correlated with the increasing number of components of MetS.

We hypothesized that the increased number of MetS components and the presence of type 2 DM could impair SAF measurements in patients with morbid obesity. In this study, we aimed to evaluate the association of MetS and its components with SAF in patients with morbid obesity and to examine whether type 2 DM or other components of MetS contributed to the increase in SAF in patients with obesity.

## 2. Methods

### 2.1. Patient’s characteristics

The data of 801 patients with morbid obesity who were volunteered for the SAF measurements during their follow-up at Marmara University Medical School Endocrinology and Metabolism Department outpatient clinic were examined retrospectively. The laboratory data [fasting plasma glucose, glycated hemoglobin (HbA1c), serum low-density lipoprotein cholesterol (LDL-c), high-density lipoprotein cholesterol (HDL-c), triglyceride, total cholesterol, creatinine] were obtained from the medical records. Patients with acute infectious and chronic inflammatory diseases, chronic kidney disease, exogenous glucocorticoid intake, concomitant alcoholism, and active smokers were excluded from the study. The classification of obesity was made using the body mass index (BMI)-dependent obesity staging of the World Health Organization (WHO). Morbid obesity was defined as a BMI over 40 kg/m^2^.

SAF measurements of 94 age-matched controls with normal BMI and normal glucose levels were examined cross-sectionally and included in the analysis. None of the control subjects had type 2 DM, hypertension, chronic renal disease, acute infectious, and chronic inflammatory diseases. All control subjects had BMI values below 30 kg/m^2^.

The study protocol was approved by the local ethics committee (09.2020.1122) and was conducted following the ethical principles stated in the Declaration of Helsinki.

#### 2.1.1. Physical measurements and laboratory evaluation

Clinical, demographic parameters (age, sex), and medical history were recorded from medical records. Height, weight, BMI, waist circumference (WC), and systolic and diastolic blood pressures were measured. BMI was calculated by dividing the weight in kg by the square of the height in m (kg/m^2^). Standing height was measured using a stadiometer nearest 0.5 cm without shoes. Bodyweight was measured using a digital electronic scale to the nearest 0.1 kg. WC was measured in the standing position in the middle of the iliac crest and the lower costal margin to the nearest 0.5 cm. Systolic and diastolic blood pressure were measured 10 min after resting in the supine position using an automated Dinamap Monitor (GE Healthcare, Freiburg, Germany).

Fasting glucose level was measured using an enzymatic ultraviolet (UV) test (hexokinase method); total cholesterol, HDL-c, and triglycerides were analyzed using an enzymatic color method on an AU5800 analyzer (Beckman Coulter, USA). Creatinine levels were analyzed using a kinetic color test (Jaffé method) with an AU5800 analyzer (Beckman Coulter, USA). HbA1c was analyzed using a boronate affinity chemistry with high-performance liquid chromatography on a Premier Hb9210 (Trinity Biotech, USA).

### 2.2. Skin autofluorescence evaluation

AGEs were measured using SAF in the forearm approximately 10 cm below the elbow fold, with an AGE Reader (DiagnOptics Technologies, Groningen, The Netherlands) at room temperature in a semidark room (Figure). Autofluorescence measurements were performed according to previous protocols [7, 21]. In brief, the AGE reader brightens approximately 4 cm^2^ skin surface with a 300 and 420 nm (peak intensity at ~ 370 nm) wavelength, and the light emitted and reflected from the skin is measured with an internal spectrometer in the range of 300–600 nm. SAF was calculated as the ratio between average emitted light intensity (420–600 nm) and the average excited light intensity (range, 300–420 nm) and multiplied by 100 and were presented as arbitrary units (AU). The SAF measurement was conducted three times, and the mean of the values was accepted as the target value.

### 2.3. Definition of metabolic syndrome and diabetes mellitus

MetS was defined according to the revised National Cholesterol Education Program-Adult Treatment Panel (NCEP-ATP) III criteria [22]. MetS present if three or more of the following five criteria were met: (1) WC over 102 cm for men and over 88 cm for women; (2) fasting triglyceride levels over 150 mg/dL or pharmacologic treatment for high triglyceride levels; (3) fasting HDL-c levels less than 50 mg/dL in women and less than 40 mg/dL in men or pharmacologic treatment for low HDL-c levels; (4) blood pressure over 130/85 mm Hg or using antihypertensive treatment; (5) fasting plasma glucose (FPG) of 100 mg/dL and higher or receiving treatment for high blood sugar, but HbA1c less than 6.5%. Patients with a previous history of type 2 DM or newly diagnosed type 2 DM were excluded from the MetS group.

Diagnosis of diabetes mellitus and prediabetes were made according to the American Diabetes Association (ADA) 2017 guidelines [23]. Subjects with FPG levels of 100–125 mg/dL but 2-h postprandial glucose levels below 140 mg/dL were defined as impaired fasting glucose (IFG). Subjects with 2-h postprandial glucose levels of 140–199 mg/dL but FPG levels below 100 mg/dL were defined as isolated impaired glucose tolerance (IGT), and subjects with FPG levels of 100–125 mg/dL and 2-h postprandial glucose levels of 140–199 mg/dL was defined as combined prediabetes (IFG+IGT).

### 2.4. Statistical analysis

Continuous variables are summarized using descriptive statistics presented as mean and standard deviation (SD). Categorical variables are summarized using counts and percentages. Categorical data were analyzed using the chi-square (χ^2^) test or Fisher’s exact test as appropriate. Student’s t-test and analysis of variance (ANOVA) were used for parametric variables. ANOVA analysis was performed to compare subjects with MetS, without MetS, and control group. After then post hoc Tukey’s test was performed to compare patients with MetS, without MetS. In addition, to compare parameters of patients with type 2 DM, patients without DM with MetS, and patients without DM without MetS first ANOVA analyses were performed.

Pearson’s correlation analysis was used to define the relationship between SAF and clinical (age, BMI, WC, systolic and diastolic blood pressure) and biochemical parameters (FPG, 2-h postprandial glucose level, HbA1c, total cholesterol, HDL-c, LDL-c, and triglycerides).

Multiple regression analysis was performed to define the predictors of SAF. Predictors with a possible influence on dependent variables were added as covariates (age, BMI, systolic blood pressure, HbA1c, LDL-c, and triglycerides).

The results were evaluated at a 95% confidence interval, and p < 0.05 was considered statistically significant. All statistical analyses were performed using software (GraphPad InStat 3.0; GraphPad Software, Inc., San Diego, CA, USA).

## 3. Results

### 3.1. General characteristics of the patients

A female preponderance [F/M: 633/168] with a mean age of 43.8 ± 10.4 years was observed. While the mean age of female subjects was 44 ± 10.5 years, it was 43.7 ± 10.4 years in males. The mean BMI was 47.8 ± 7.4 kg/m^2^ and 47.9 ± 7.9 kg/m^2^ in female and male patients, respectively. Type 2 DM and hypertension were observed in 40.9% (n = 328), 43.7% (n = 357) of the patients, respectively. The prevalence of MetS was 65.5% (n = 525), 79% (n = 416) of them was female and 21% (n = 109) was male subjects. On the other hand, 78.6% (217) of the 276 subjects without MetS were female and 21.4% (59) male. Clinical characteristics and laboratory parameters are shown in Table 1. Statistically significant differences between obese patients and the control group were detected in FPG, HbA1c, triglyceride, LDL-c, HDL-c, and total cholesterol levels (p < 0.001).

### 3.2. Skin autofluorescence evaluation according to the presence of MetS

When patients with obesity were divided into two subgroups as patients with and without MetS, there was a significant difference in systolic (p < 0.001) and diastolic blood pressure (p = 0.001), FPG (p < 0.001), HbA1c (p = 0.001), total cholesterol (p = 0.007), triglyceride (p = 0.019), but SAF measurements did not differ according to the presence of MetS (p = 0.091). By contrast, patients with MetS had higher SAF measurements than the control group (p = 0.034), but the SAF measurements of patients without MetS were not statistically different from the control group (p = 0.076).

When patients were grouped according to the number of MetS components, there were 86 (10.7%) patients with one criterion, 190 (23.7%) patients with two criteria, 301 (37.6%) patients with three criteria, 172 (21.5%) patients with four criteria, and 52 (6.5%) patients with five criteria. The levels of SAF according to the number of MetS components were as follows: 1.86 ± 0.37 AU, 1.78 ± 0.35 AU, 1.83 ± 0.42 AU, 1.87 ± 0.48 AU, and 2.09 ± 0.59 AU, respectively. Although patients with five components of MetS had higher SAF levels (p = 0.019), there was no statistical difference between the other groups.

### 3.3. Skin autofluorescence evaluation according to glucose metabolism

After excluding individuals with type 2 DM from among patients with obesity, subjects without DM were categorized into two subgroups as those with and without MetS. The findings of patients were compared in three groups; patients with type 2 DM, patients without DM with MetS, and patients without DM without MetS. General characteristics and biochemical parameters according to these subgroup analyses are summarized in Table 2. In patients with obesity, there was no statistical difference in SAF measurements in patients with or without type 2 DM (p = 0.272). After excluding patients with type 2 DM, there was still no difference in SAF measurements in patients without DM with MetS, and patients without DM without MetS (p = 0.213).

When the patients were divided into three subgroups as patients with type 2 DM (n = 328), impaired glucose metabolism (n = 240), and normal glucose metabolism (n = 233), SAF measurements were found to be similar: 1.87 ± 0.46 AU, 1.84 ± 0.45 AU, and 1.84 ± 0.40 AU (p = 0.513).

### 3.4. Correlation and multiple regression analyses

A weak positive correlation with SAF and systolic blood pressure (r: 0.096, p = 0.009) was observed. There was no significant correlation with SAF and other parameters (age, BMI, WC, FPG, 2-h postprandial glucose level, HbA1c, total cholesterol, HDL-c, LDL-c, and triglycerides) (Table 3).

Multiple regression analysis showed that SAF was independently associated with systolic blood pressure (p = 0.023) and HbA1c (p < 0.001), but there were no association between SAF and age, BMI, LDL-c, and triglycerides (R^2^ = 4.22%, p = 0.005) (Table 4).

## 4. Discussion

In this study, patients with morbid obesity and those with MetS had higher SAF measurements than healthy controls. However, they found no difference in SAF levels between patients with and without MetS. There was no difference in patients with obesity between SAF levels according to type 2 DM or impaired glucose metabolism. Although MetS and type 2 DM are known as factors related to increased SAF levels, obesity was related to elevated SAF measurements independent of concomitant comorbidities.

Uribarri et al. study, which included subjects with central obesity, reported that higher serum AGEs were present in subjects with more than one MetS criterion compared with subjects without MetS criteria [13]. In van Waateringe et al. [19] and Den Engelsen et al.’s [3] studies, higher SAF measurements were detected in subjects with MetS compared with patients without MetS. Although there was no difference in SAF measurements according to the presence of MetS in our study, it could be detected if more patients with morbid obesity without MetS were included. However, due to the relatively fewer patients with morbid obesity without MetS, it is difficult to increase the number of these patients. In addition, previous studies mostly included overweight subjects or patients with class 1 and rarely class 2 obesity, but we only evaluated patients with morbid obesity, and these differences between patient groups may be responsible for these divergent results.

Several previous reports, which evaluated serum AGEs, showed that serum AGEs were not increased in individuals with obesity without MetS characteristics [24,25]. On the other hand, van Waateringe et al.’s study showed that patients with MetS had similar SAF levels whether obese or not, and there was also no statistically significant difference in SAF between individuals with and without obesity, but patients with enlarged WC had higher SAF levels [19]. In our study, there was no statistical difference in SAF levels between patients with obesity without MetS and the control group, but when all patients with obesity were compared with nonobese subjects, SAF levels were found to be higher in the obese group compared with controls, but SAF levels did not differ in patients with or without MetS. Maybe we can attribute this due to the presence of enlarged WC in all our patients with obesity.

In Tanaka et al.’s study with patients with type 2 DM, systolic blood pressure was related to SAF [26], and in van Waateringe et al.’s study with subjects with MetS elevated blood pressure was related to higher SAF [19]. In our study, multivariate regression analysis showed that systolic blood pressure explained 2.7% of SAF measurements, but no other parameters were statistically significant.

Increased AGEs levels in the tissue and circulation are well-established in patients with diabetes [9,11]. Although we hypothesized that the presence of type 2 DM could also affect SAF in patients with morbid obesity, there was no difference in SAF measurement between patients with and without type 2 DM with obesity. However, when we compared patients with type 2 DM with the controls, SAF was higher in patients with type 2 DM. In previous reports, obesity-related subclinical atherosclerosis and endothelial dysfunction [27] have been reported regardless of concomitant diabetes and metabolic syndrome [28]. We may attribute these findings to the effect of obesity itself. Morbid obesity could be the main cause of increased SAF levels rather than concomitant type 2 DM or MetS.

In Sprung et al.’s study, which used flow-mediated dilatation (FMD) for the evaluation of endothelial dysfunction, patients with MetS also had lower FMD compared with patients without MetS, both in the obese and nonobese group [29], and also an increased number of MetS components was found to be associated with a lower FMD. He et al. showed a relationship between plasma receptor of AGEs levels and MetS and its components in adolescents with central obesity [20]. In van Waateringe et al.’s study, as the components of MetS increased, higher SAF levels were detected [19]. In our study, patients with five components of MetS had higher SAF levels than other groups.

The major limitation of this study is the lack of diet, smoking, and exercise history, which can influence AGEs levels and SAF measurements. Also, this study is performed retrospectively, which makes it difficult to understand the reasons for the changes in SAF levels. To define causality in the relationship between SAF levels and morbid obesity, type 2 DM, and MetS, long-term follow-up studies including serum AGEs, and SAF measurements with the evaluation of a diet diary and smoking habits are needed.

In conclusion, this study showed that morbid obesity was the main cause of increased SAF levels rather than concomitant type 2 DM or MetS. Although MetS and type 2 DM are known as factors related to increased SAF, obesity can cause elevated SAF measurements in different ways independent of concomitant comorbid diseases. Larger studies with longer follow-up are needed to enlighten the underlying mechanism.

## Figures and Tables

**Figure:**
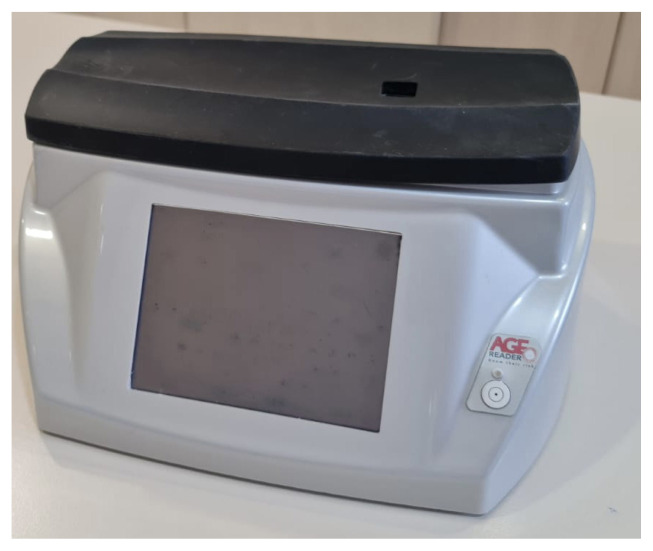
The image of the AGE reader used in the evaluation of skin autofluorescence.

**Table 1:** Clinical characteristics and laboratory findings of the patients with obesity and control group.

Parameter	All n = 801	MetS + n = 525	MetS − n = 276	Control n = 94	p
**Age (years)**	43.8 ± 10.4	45.6 ± 10.4	40.5 ± 9.7	44.0 ± 11.0	**<0.001**
**Sex (F/M)**	633/168	416/109	217/59	66/28	0.640
**DM, n (%)**	328 (40.9)	286 (54.5)	42 (15.2)	0 (0)	**<0.001** ¥
**Hypertension, n (%)**	350 (43.7)	300 (57.1)	50 (18.1)	0 (0)	**<0.001** a
**BMI (kg/m** **^2^** **)**	47.8 ± 7.4	48.1 ± 7.5	46.8 ± 7.1	24.7 ± 3.4	**<0.001**
**Weight (kg)**	127.6 ± 22.3	128.4 ± 22.1	126.1 ± 22.7	67.4 ± 11.1	**<0.001**
**WC of women (cm)**	127.2 ± 14.2	127.9 ± 14.9	125.7 ± 12.8	86.8 ± 9.7	**<0.001**
**WC of men (cm)**	134.9 ± 15.3	136.5 ± 14.4	131.3 ± 16.9	88.3 ± 6.5	**<0.001**
**SBP (mmHg)**	128.5 ± 20.8	131.8 ± 21.3	121.9 ± 18.0	108.7 ± 12.5	**<0.001** γ
**DBP (mmHg)**	78.7 ± 14.8	80.0 ± 15.6	76.0 ± 12.7	66.2 ± 9.4	**<0.001** δ
**FPG (mg/dL)**	96.5 ± 26.4	98.7 ± 26.1	92.8 ± 26.6	90.9 ± 18.6	**<0.001** β
**HbA1c (%)**	6.0 ± 1.5	6.1 ± 1.6	5.8 ± 1.2	5.0 ± 0.6	**<0.001** §
**Triglycerides (mg/dL)**	160.7 ± 89.6	167.0 ± 97.9	148.2 ± 68.6	98.5 ± 75.2	**<0.001**
**HDL-c (mg/dL)**	46.6 ± 10.3	46.7 ± 10.2	46.3 ± 10.4	53.7 ± 14.1	**<0.001** #
**LDL-c (mg/dL)**	130.3 ± 40.2	132.2 ± 42.6	126.7 ± 35.0	115.7 ± 37.3	**0.003**
**SAF (AU)**	1.85 ± 0.44	1.87 ± 0.46	1.81 ± 0.38	1.72 ± 0.30	**0.016** *

Values reported as mean ± standard deviation.

BMI, Body mass index; DBP, diastolic blood pressure; DM, type 2 diabetes mellitus; F, female; FPG, fasting plasma glucose (65–100 mg/dL); HDL, high-density cholesterol (35–70 mg/dL); LDL, low-density cholesterol (0–140 mg/dL); M, male; MetS, metabolic syndrome; SAF, skin autofluorescence; SBP, systolic blood pressure; Total-C, total cholesterol (30–200 mg/dL); triglycerides (30–200 mg/dL); WC, waist circumference.

¥ p < 0.001 for MetS+ vs. MetS−;

a p < 0.001 for MetS+ vs. MetS;

γ p < 0.001 for MetS+ vs. MetS−;

δ p = 0.001 for MetS+ vs. MetS−;

β p < 0.001 for MetS+ vs. MetS−;

§ p = 0.001 for MetS+ vs. MetS−;

# p = 0.019 for MetS+ vs. MetS−. There was no statistical significance among other parameters between MetS+ vs. MetS− groups.

* p = 0.091 for MetS+ vs. MetS−; p = 0.034 for MetS+ vs. control group; p = 0.076 for MetS− vs. control group

**Table 2:** Clinical characteristics and laboratory findings of patients according to the presence of diabetes mellitus and metabolic syndrome.

Parameter	DM n = 328	MetS + n = 239	MetS − n = 234	p
**Age (years)**	46.9 ± 10.1	43.3 ± 9.9	39.8 ± 9.9	**<0.001**
**Sex (F/M)**	255/73	190/49	188/46	0.679
**BMI (kg/m** **^2^** **)**	48.3 ± 7.7	48.3 ± 7.6	46.4 ± 6.5	0.071
**Weight (kg)**	127.3 ± 23.1	129.5 ± 21.9	126.2 ± 21.5	0.401
**WC of women (cm)**	128.3 ± 12.0	127.3 ± 17.7	125.3 ± 12.9	0.071
**WC of men (cm)**	137.0 ± 11.7	137.7 ± 10.4	130.9 ± 17.6	0.121
**SBP (mmHg)**	130.5 ± 20.4	131.3 ± 22.2	122.5 ± 18.7	**<0.001**
**DBP (mmHg)**	79.1 ± 15.0	80.2 ± 15.8	76.3 ± 13.0	0.060
**FPG (mg/dL)**	113.9 ± 52.9	93.6 ± 13.2	88.2 ± 10.1	**<0.001**
**HbA1c (%)**	6.3 ± 1.4	5.4 ± 0.5	5.4 ± 0.4	**<0.001**
**Triglycerides (mg/dL)**	167.1 ± 83.5	159.6 ± 95.2	147.9 ± 67.4	**0.033**
**HDL-c (mg/dL)**	46.2 ± 10.8	47.2 ± 12.7	47.1 ± 10.3	0.261
**LDL-c (mg/dL)**	131.0 ± 37.9	132.6 ± 46.8	127.0 ± 35.9	0.348
**SAF (AU)**	1.85 ± 0.42	1.86 ± 0.43	1.82 ± 0.39	0.272*

Values reported as mean ± standard deviation.

BMI, Body mass index; DBP, diastolic blood pressure; DM, type 2 diabetes mellitus; F, female; FPG, fasting plasma glucose (65–100mg/dL); HDL, high-density cholesterol (35–70 mg/dL); LDL, low-density cholesterol (0–140 mg/dL); M, male; MetS, metabolic syndrome; SAF, skin autofluorescence; SBP, systolic blood pressure; Total-C, total cholesterol (30–200 mg/dL); triglycerides (30–200 mg/dL); WC, waist circumference.

* p = 0.911 for DM+ vs. MetS+; p = 0.123 for DM+ vs. MetS−; p = 0.213 for MetS+ vs. MetS−

**Table 3:** Correlation analysis of parameters with skin autofluorescence.

	R	p value
**Age (years)**	0.041	0.263
**BMI (kg/m** **^2^** **)**	0.029	0.420
**WC**	0.020	0.600
**SBP (mmHg)**	0.096	**0.009**
**DBP (mmHg)**	0.041	0.257
**HbA1c (%)**	0.039	0.299
**Triglycerides (mg/dL)**	0.037	0.302
**HDL-c (mg/dL)**	0.054	0.137
**LDL-c (mg/dL)**	0.014	0.700

BMI, Body mass index; DBP, diastolic blood pressure; HDL, high-density cholesterol; LDL, low-density cholesterol; SBP, systolic blood pressure; WC, waist circumference.

**Table 4:** Multiple regression analysis of parameters associated with skin autofluorescence.

Dependent variable	Independent variable	Odds ratio (%)	Standard error	%95 CI	p value	Pseudo R^2^
**SAF**	**Age (years)**	1.090	0.062	1.001–1.007	0.313	4.22%
	**BMI (kg/m** **^2^** **)**	0.301	0.005	0.995–1.007	0.519	
	**SBP (mmHg)**	0.413	0.002	1.003–1.004	**0.023**	
	**HbA1c (%)**	0.454	0.008	1.002–1.011	**<0.001**	
	**Triglycerides (mg/dL)**	0.560	0.001	1.001–1.026	0.957	
	**LDL-c (mg/dL)**	2.907	0.001	1.000–1.015	0.600	

BMI, Body mass index; LDL, low-density cholesterol; SAF, skin autofluorescence; SBP, systolic blood pressure

## Data Availability

The author elects to not share the data.
